# Enhancing recovery and sensitivity studies in an unconventional tight gas condensate reservoir

**DOI:** 10.1007/s12182-018-0220-7

**Published:** 2018-03-27

**Authors:** Min Wang, Shengnan Chen, Menglu Lin

**Affiliations:** Chemical and Petroleum Engineering Department, University of Calgary, 2500 University Drive NW, Calgary, AB T2N1N4 Canada

**Keywords:** Tight gas condensate reservoirs, Enhanced/improved gas recovery, Produced gas injection, Sensitivity study, Economic benefit

## Abstract

The recovery factor from tight gas reservoirs is typically less than 15%, even with multistage hydraulic fracturing stimulation. Such low recovery is exacerbated in tight gas condensate reservoirs, where the depletion of gas leaves the valuable condensate behind. In this paper, three enhanced gas recovery (EGR) methods including produced gas injection, CO_2_ injection and water injection are investigated to increase the well productivity for a tight gas condensate reservoir in the Montney Formation, Canada. The production performance of the three EGR methods is compared and their economic feasibility is evaluated. Sensitivity analysis of the key factors such as primary production duration, bottom-hole pressures, and fracture conductivity is conducted and their effects on the well production performance are analyzed. Results show that, compared with the simple depletion method, both the cumulative gas and condensate production increase with fluids injected. Produced gas injection leads to both a higher gas and condensate production compared with those of the CO_2_ injection, while waterflooding suffers from injection difficulty and the corresponding low sweep efficiency. Meanwhile, the injection cost is lower for the produced gas injection due to the on-site available gas source and minimal transport costs, gaining more economic benefits than the other EGR methods.

## Introduction

The successful application of horizontal drilling and multistage hydraulic fracturing technologies has boosted oil and gas production from tight reservoirs in the last decade. Although commercial development is enabled by the advanced technologies, estimated primary recovery factors remain to be as low as 5%–15%, owing to the ultra-low permeability (Hoffman 2012). Currently, a liquid-rich tight gas reservoir (e.g., Montney Formation) has attracted interest (Cui et al. 2013; Rivard et al. 2014). In a gas condensate reservoir, reservoir fluids appear as gas phase under initial conditions. With the depressurization of the reservoir during the primary production, liquid condenses from the gas phase and builds up once the in situ reservoir pressure drops below the dew-point pressure, especially around the fractures and the well bottom hole. The condensate liquid will not flow until a critical condensate saturation is achieved. It is generally accepted that three zones are present in the formation from the wellbore to the reservoir boundary: (1) mobile gas and mobile condensate region near the wellbore, (2) transition zone including mobile gas and immobile oil, and (3) gas phase zone without condensate dropout (Penuela and Civan 2000). The trapped condensate typically cannot flow, leaving a large amount of high-quality oil unproduced in the reservoir. Nevertheless, gas production will decrease as the presence of condensate restricts the gas flow toward the wellbore (Hinchman and Barree 1985; Moses and Donohoe 1987; Vo et al. 1989; Li and Firoozabadi 2000; Pope et al. 2000). Meanwhile, the condensate blockage problem is exacerbated significantly by the ultra-low reservoir permeability in a tight gas condensate reservoir, and the gas production rate could decrease by 50%–80% within the first 2 years (Ayyalasomayajula et al. 2005). Thus, it is important to investigate the potential performance of the EGR methods to alleviate condensate blockage and further increase the recovery factor in tight gas condensate reservoirs. Extensive study has been performed to maintain the reservoir pressure above the dew-point pressure in conventional gas condensate reservoirs, using techniques such as water injection (Matthews et al. 1988), lean gas injection (Smith and Yarborough 1968; Abel et al. 1970; Sigmund and Cameron 1977; Abasov et al. 2000), CO_2_ injection (Goricnik et al. 1995; Narinesingh and Alexander 2014) and N_2_ injection (Aziz 1983; Abdulwahab and Belhaj 2010; Sadooni and Zonnouri 2015). Usually, two schemes of fluid injection are employed for pressure maintenance in a condensate reservoir. One is full pressure maintenance where the fluid is continuously injected into the reservoir, while at the same time, the condensate is produced. The other is the partial pressure maintenance where gas is injected into the reservoir after the primary depletion to slow pressure decline and re-vaporize the condensate (Abel et al. 1970; Meng and Sheng 2016). Currently, researchers mainly rely on laboratory studies or reservoir simulations to investigate the performance of EGR methods in unconventional tight reservoirs due to the lack of field test data. Yu et al. (2014) studied the efficiency of CO_2_ injection to enhance the gas recovery in a shale gas reservoir, considering the adsorption of CO_2_ in the shales with a high total organic content. An experimental design method was employed to search for the best operational scenario for the CO_2_ injection. Sheng (2015a, b) investigated the huff-n-puff performance of the produced gas in a shale gas condensate reservoir via a simplified simulation model containing only one fracture stage. They concluded that huff-n-puff methane injection is an effective option to enhance the gas and oil recovery for a shale gas reservoir with a permeability of 100 nD (i.e., 0.0001 mD). Haghshenas et al. (2017) simulated CO_2_ huff-n-puff in a liquid-rich Canadian unconventional reservoir accounting for the fluid adsorption and the compositional heterogeneity. CO_2_ huff-n-puff results were only positive for certain operating conditions, and additional sensitivity study to EGR operations was needed. The mechanism and feasibility of the EGR is still not very clear, and it is necessary to investigate the key factors affecting the effectiveness in the tight gas condensate reservoir.

In this paper, we focus on evaluating different EGR methods after the reservoir has been depleted for several years and the flowing bottom-hole pressure of producers remains below the dew-point pressure. A sensitivity study was further conducted on the key factors that affect the performance of EGR methods. More specifically, a geological model which contains 27 horizontal wells was built and a sub-model containing 3 wells was cut out for the reservoir simulation, each well containing nearly 30 stages of hydraulic fractures. Three EGR methods including produced gas flooding, CO_2_ flooding, and waterflooding were then applied and their performances were evaluated. Sensitivity studies of the key operational and geological parameters were conducted to investigate their effects on the gas and condensate production. Economic feasibility of EGR methods was also analyzed. This work can advance the understanding of the mechanisms of enhancing recovery in unconventional tight condensate gas reservoirs and provide a reference for the future EGR field application in the Montney Formation.

## Geological model

**Fig. 1:**
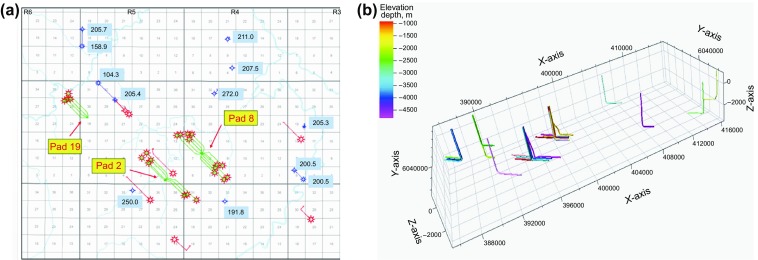
Well locations in Accumap (numbers in black indicating the formation thickness at the well location) (**a**) and the geological model (**b**)

The target reservoir, located in the Montney play, is situated at the border of Alberta and British Columbia, in the Western Canadian Sedimentary Basin, Canada. The Montney Formation is composed of two production zones: the upper Montney and the lower Montney, containing 449 trillion cubic feet of marketable natural gas, 14,521 million barrels of marketable natural gas liquids and 1125 million barrels of oil, as estimated by Canada’s National Energy Board (NEB 2013). Multistage hydraulic fractures placed along a horizontal well are the main completion method in the Montney Formation to achieve commercial well production rates (Kuppe et al. 2012). In this study, a geologic model, covering an area of 34,000 m long and 18,000 m wide, is built for a liquid-rich gas play in the Montney Formation. Reservoir depth ranges from 2800 to 3500 m with a thickness of 200 m. Twenty-seven horizontal wells have been drilled and fractured in the simulated area, and their locations are shown in Fig. 1a, and Fig. 1b shows their positions in the geological model. Reservoir properties such as matrix permeability, porosity and water saturation are derived using laboratory measurements and well-logging data (Ghanizadeh et al. 2014). The reservoir properties such as permeability and porosity are listed in Table 1.

**Table 1:** Reservoir model properties

Parameters	Value
Reservoir temperature, °C	98
Reservoir pressure, MPa	30.5
Matrix permeability, mD	0.004–0.009
Matrix porosity	0.02–0.09
Matrix water saturation	0.3

## Reservoir simulation model

### Model description

**Fig. 2:**
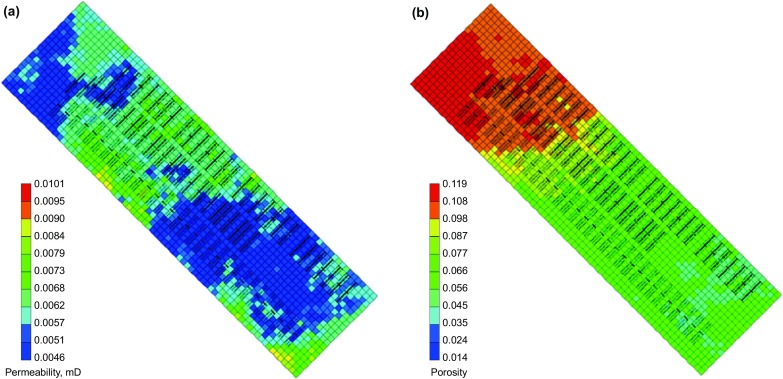
Permeability and porosity variation of the simulation model. **a** Permeability distribution. **b** Porosity distribution

**Fig. 3:**
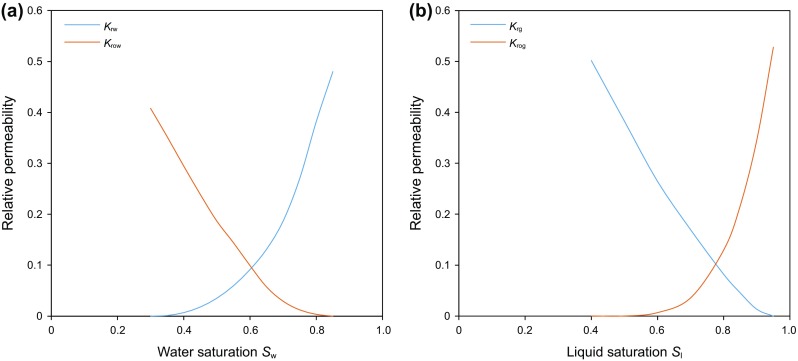
Relative permeability curves for the Montney Formation. **a** Oil and water relative permeability curves (*k*_rw_: water relative permeability; *k*_row_: oil relative permeability) **b** Liquid and gas relative permeability curves (*k*_rg_: gas relative permeability; *k*_rog_: oil relative permeatility)

The geological model was upscaled, and a section of the model containing three horizontal wells was selected and history matched for the reservoir simulation studies. All three wells are hydraulically fractured. Well 1 and Well 3 are both 3000 meters long with 31 stages, while Well 2, which is located in the middle, is 2100 meters with 27 stages. Properties of the hydraulic fractures are shown in Table 2, and the perforation type is open hole. The reservoir model dimension is 1050 m wide with 21 grids in the I direction, 3800 m long with 76 grids in the J direction and 60 m thick with 7 grids in the K direction. Porosity and permeability distributions of the numerical model are shown in Fig. 2. Local refining grids were generated to represent the hydraulic fractures in the reservoir model. Relative permeability curves of the reservoir matrix are shown in Fig. 3 (Lan et al. 2015). For the hydraulic fractures, the relative permeability curves are assumed to be two straight lines. For the multiphase fluid flow in the reservoir, the three phase permeabilites are calculated by Stone’s second model (CMG 2016).

**Table 2:** Properties of hydraulic fractures

Parameters	Value
Hydraulic fracture half-length, m	125
Hydraulic fracture height, m	40
Hydraulic fracture conductivity, Darcy × mm	64
Hydraulic fracture spacing, m	80

### Reservoir fluid properties

**Fig. 4:**
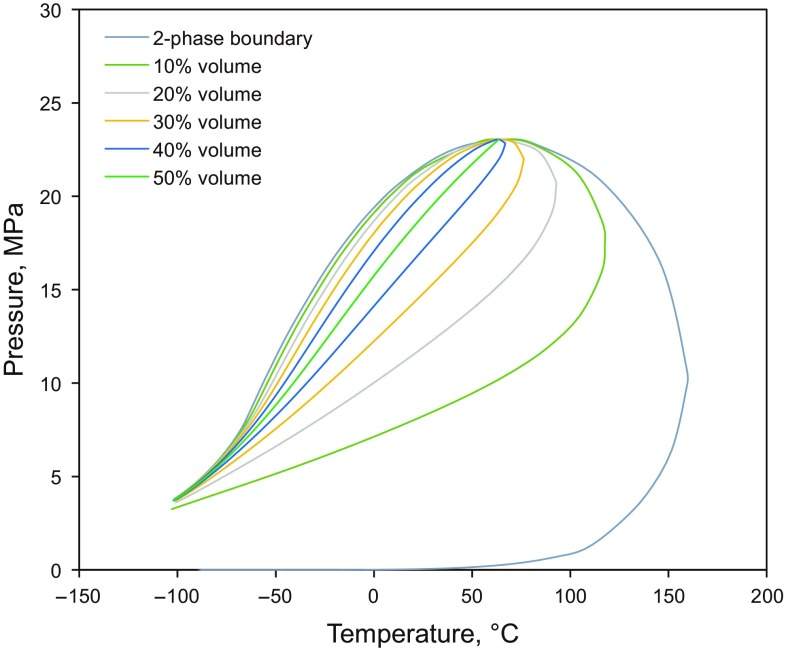
Phase behavior diagram of the reservoir fluids

The area of interest is located in a gas condensate zone. Figure 4 depicts the calculated phase envelope of the recombined fluid at a production gas–oil ratio of 1200 m^3^/m^3^. It can be seen that the dew-point temperature is 64 °C and the dew-point pressure is 23.2 MPa. Reservoir conditions (98 °C, 30.5 MPa) belong to the retrograde condensation area of the generated phase envelope, as seen in the figure.

Reservoir pressure decreases as the well production proceeds, while reservoir temperature keeps constant. When the pressure drops below the dew-point pressure, the liquid condensate begins to condense from the gas phase and remains immobile till its saturation reaches a critical value. The newly formed liquid will not only reduce the amount of condensate (i.e., oil) production at the wellhead but also block the gas from flowing toward the wellbore, which may reduce the gas production rate at the same time. Thus, it is essential to maintain average reservoir pressure above the dew-point pressure and slow further pressure decline when developing gas condensate reservoirs.

### History matching studies

**Fig. 5:**
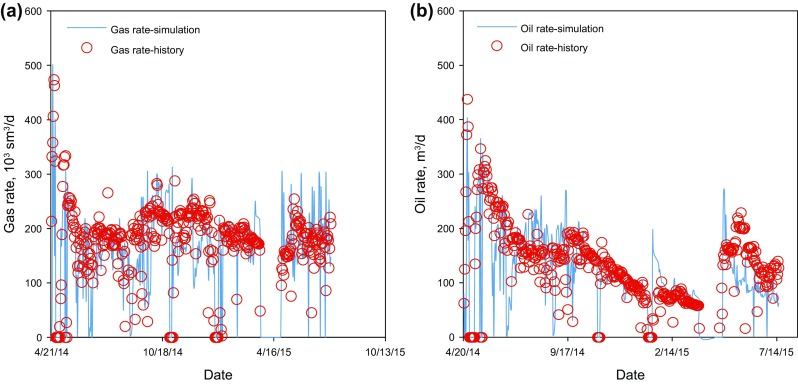
History matching results for Well 3. **a** Gas rates. **b** Condensate (oil) rates

History matching was performed to further tune the reservoir simulation model to better represent the formation rock and fluid properties. In this model, the bottom-hole pressures of the producers were applied as constraints, while the gas and condensate production rates were matched. Reasonable history matching results were achieved for all three wells, and Fig. 5 depicts the history matching results for Well 3. As seen, a production history of 450 days has been history matched, and the tuned model was reliable for reservoir simulations and production predictions.

## Results and discussion

**Fig. 6:**
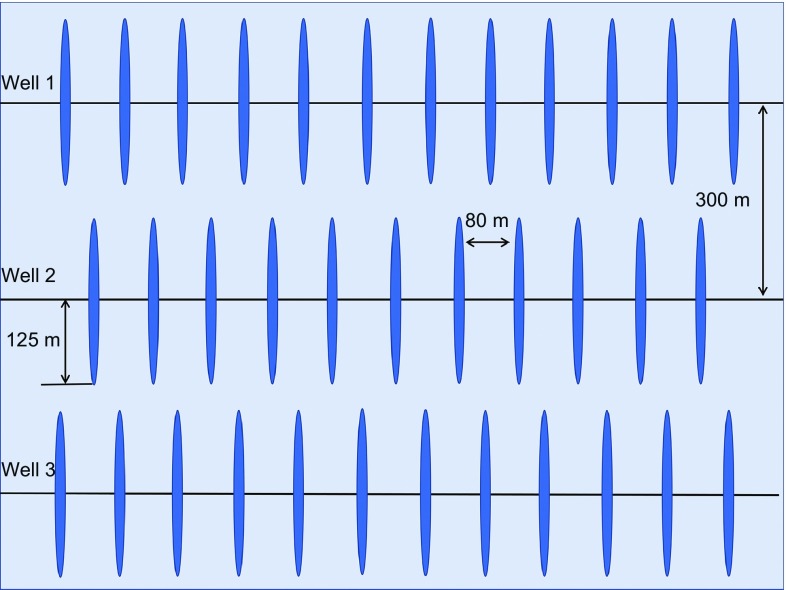
Schematic diagram of the three fractured horizontal wells and the simulation area

Figure 6 depicts the schematic diagram demonstrating the three hydraulic fractured horizontal wells that are distributed in the simulation model. The well spacing is 300 m, the fracture spacing is 80 m, and the half-length of the hydraulic fracture is 125 m. Primary production continues for about 5 years (from Day 450 to Day 2200) and results suggest that the average reservoir pressure drops to 22.9 MPa, which is slightly lower than the dew-point pressure at 23.2 MPa (See Fig. 8). The aforementioned three EGR methods are then applied on Day 2200 to prevent a large amount of liquid being condensed from the gas phase. Only primary production is applied in the base case. Scenario 1 represents the produced gas flooding scenario, where Well 2 is converted to a produced gas injector, while Well 1 and Well 3 remain producers after 5 years of primary production. It should be noted that Well 2 is converted back to a producer after injecting produced gas for ten years. Scenarios 2 and 3 are the CO_2_ flooding scenario and the waterflooding scenario. Similarly, Well 2 is converted to a CO_2_ injector or water injector, while Well 1 and Well 3 still remain producers after 5 years of depletion. The water injection scenario is included in the simulations only for the comparison with gas injection scenarios.

### Reservoir pressure

**Fig. 7:**
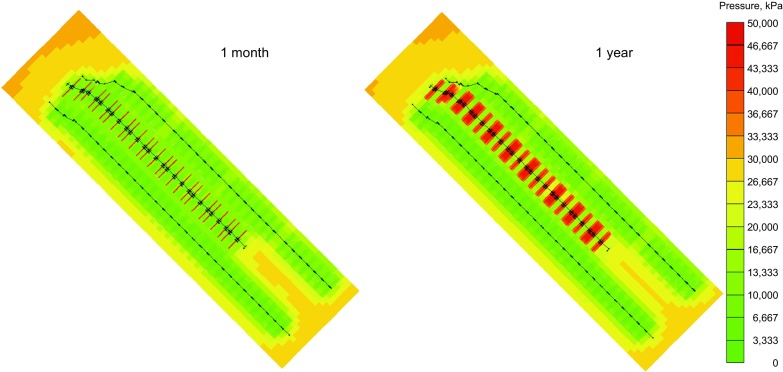
Pressure distribution for the CO_2_ injection scenario (unit kPa)

**Fig. 8:**
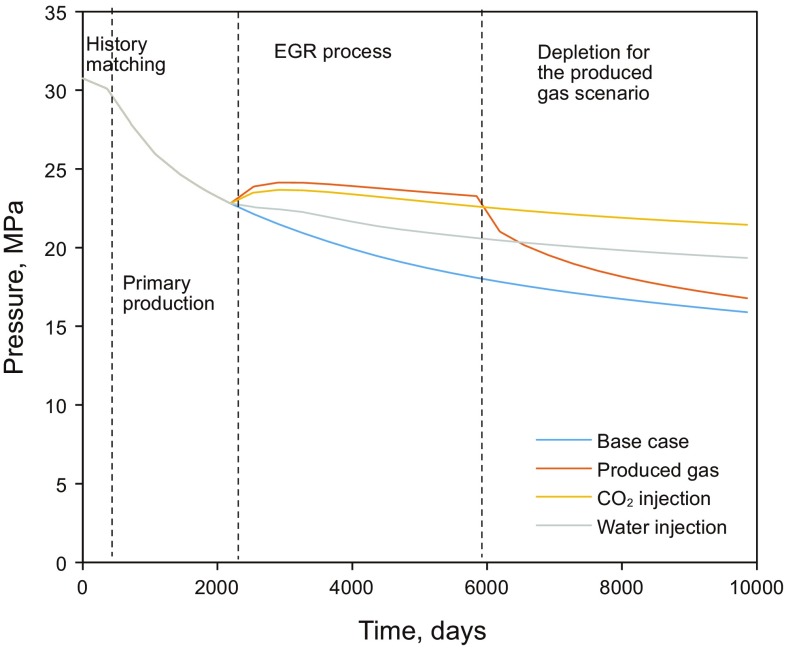
Impact of the fluid injection on the average reservoir pressure

As mentioned above, pressure maintenance is essential for a gas condensate reservoir. The flooding characteristics in tight reservoirs are different from those in the conventional reservoirs. Figure 7 shows the pressure distribution of the CO_2_ flooding scenario after 1 month and 1 year’s injection, respectively. We can observe that the injection gas first flows in the hydraulic fractures and then penetrates into the surrounding matrix under the high injection pressure. High pressure is still mainly limited in the areas near the hydraulic fractures after 1 year injection due to the low matrix permeability. The reservoir pressure for each scenario following the primary production is also depicted in Fig. 8. As seen, the average reservoir pressure keeps decreasing in the base case where no fluid injection is applied. The average reservoir pressures for the produced gas injection and CO_2_ injection scenarios are both above the dew-point pressure while that of the waterflooding scenario slightly increases, but fails to stay above the dew-point pressure. This is because the ultra-low permeability of the reservoir matrix (0.004–0.009 mD) restricts the water penetration into the formation rocks, leading to a low water sweep efficiency. In addition, the higher sweep efficiency of the gas injection can also be attributed to the following aspects: (1) the condensate oil swells and its viscosity decreases due to the gas dissolution in oil; and (2) the interfacial tension could be reduced or eliminated if a miscible condition is reached. However, due to the low permeability, the high pressure area only remains near injectors and pressure around producers is still low, which lowers the positive effect of viscosity and interfacial tension reduction. In the produced gas injection scenario, much injected gas accumulates near the injector during the flooding process. In order to recover the large amount of injected gas for better revenue, Well 2 is converted back to a producer after 10 years of produced gas flooding. The reservoir pressure then drops significantly.

### Production performance of EGR methods

**Fig. 9:**
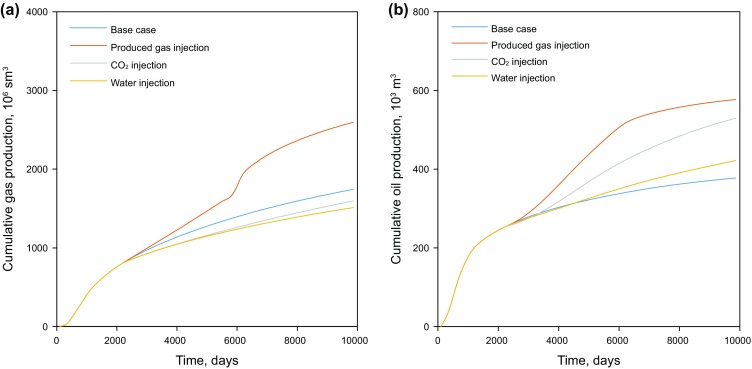
Cumulative gas and condensate production of the three EGR methods. **a** Cumulative gas production measured under standard condition (sm^3^). **b** Cumulative condensate (oil) production

The injection pressure for the three scenarios is set the same at 45 MPa. Figure 9 shows the cumulative gas and condensate production of the produced gas injection, CO_2_ injection and water injection for the target formation. It should be noted that for the produced gas injection scenario the amount of produced gas that is injected into the formation needs to be subtracted from the total gas production in order to calculate the net natural gas production, which is shown in Table 3. It can be seen from Fig. 9a and Table 3 that the base case leads to the highest gas production, followed by CO_2_ injection, while the produced gas injection and water injection scenarios share a low cumulative gas production. However, the produced gas injection and CO_2_ injection display a significant increase in the cumulative condensate production. The cumulative condensate production of the produced gas injection is 52.7% higher than that of the base case and CO_2_ injection indicates a 40.0% improvement in cumulative condensate production (see Fig. 9b and Table 3). Although its gas production is reduced, the water injection scenario also demonstrates a slightly higher cumulative condensate production than those of the base case scenario. This is because the injected water reduces the relative permeability of the gas phase and thus decreases the gas ability to flow to the wellbore. However, the reservoir pressure increases, preventing liquid being condensed in the reservoir.

**Table 3:** Cumulative production for different enhanced/improved gas methods

	Produced gas injection	CO_2_ injection	Waterflooding	Base case
Cumulative injected volume, m^3^	1.00 × 10^9^	9.38 × 10^8^	8.52 × 10^5^	0
Net cumulative gas production, m^3^	1.56 × 10^9^	1.67 × 10^9^	1.57 × 10^9^	1.72 × 10^9^
Cumulative condensate production, m^3^	5.74 × 10^5^	4.83 × 10^5^	4.30 × 10^5^	3.75 × 10^5^
BOE	1.31 × 10^7^	1.32 × 10^7^	1.23 × 10^7^	1.28 × 10^7^

As aforementioned, Well 2 is converted back into a producer and is put into production on Day 5850. It can be seen that a large amount of gas is produced during this stage shown as sharp increases in the gas production for the produced gas injection scenario in Fig. 9a. It is worth pointing out that the cumulative gas condensate production (i.e., cumulative oil production) only slightly increases during such process due to a low percentage of the heavy components in the injected gas near the injector.

The barrel of oil equivalent (BOE) is adopted to assess the gas and condensate productivity for the different scenarios. The BOE is an industrial unit of energy equivalent to the amount of energy released by burning one barrel of crude oil. The calculated BOE results are shown in Table 3. The produced gas injection displays the highest BOE amount, followed by the CO_2_ injection, base case and waterflooding, respectively. In addition, the BOE of the waterflooding is lower than that of the base case. This is because the injected water has decreased the effective gas permeability in the formation, leading to a lower gas production rate. In other words, the increase in condensate production due to a higher reservoir pressure during waterflooding cannot compensate for the loss of gas production compared with the base case. The cumulative condensate production of the base case is the lowest among all scenarios, as the low reservoir pressure of the base case promotes condensate to be condensed and left unproduced in the reservoir.

### Phase envelop change

**Fig. 10:**
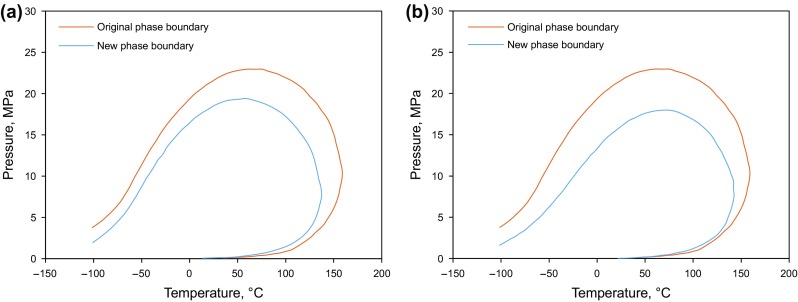
Phase diagrams of the produced gas injection and CO_2_ injection scenarios. **a** *P*–*T* diagram with produced gas injection. **b** *P*–*T* diagram with CO_2_ injection

The phase diagram changes during the produced gas injection and CO_2_ injection processes as a result of the compositional change of the reservoir fluids. Figure 10 demonstrates the new phase diagram with the production gas–oil ratio of 1500 with produced gas or CO_2_ injection. It can be seen that both the critical pressure and temperature decrease, and the two-phase region shifts to the left side, compared to the phase envelope shown in Fig. 4. Such changes will help prevent the oil condensation in the formation under the reservoir conditions and further increase the condensate production at the wellhead.

### Net present value

Besides the BOE, the net present value of the EGR processes has been estimated via the following equation (Yu and Sepehrnoori 2014):

1 $${{NPV}} = \mathop \sum \limits_{j = 1}^{n} \frac{{(V_{\text{F}} - C_{\text{F}} )_{j} }}{{\left( {1 + i} \right)^{j} }} - \left[ {{{FC}} + \mathop \sum \limits_{k = 1}^{N} \left( {C_{\text{well}} + C_{\text{fracture}} } \right)} \right]$$

where $V_{\text{F}}$ is the related revenue due to the fluid injected, *C*_F_ is all the related cost due to the fluid injection, $C_{\text{well}}$ and $C_{\text{fracture}}$ are the costs of the well drilling and completion, *N* is the number of horizontal wells, *n* is the number of years, and *i* is the discount rate or interest rate, *FC* summarizes all the other cost, such as cost related to well-type conversion and operations. There is no well-type conversion cost for the base case.

$C_{\text{well}}$, $C_{\text{fracture}}$, *N*, *n* and *i* are all the same for the base case and the three EGR scenarios. Assuming the *FC* is also constant, and then Eq. (1) can be simplified to:

2 $${{NPV}} = \mathop \sum \limits_{j = 1}^{n} \frac{{(V_{\text{oil}} + V_{\text{gas}} - C_{\text{F}} )_{j} }}{{\left( {1 + i} \right)^{j} }} - C$$

where *V*_gas_ and *V*_oil_ are the annual gas and oil revenue.  In this study, a gas price of $3.0/Mcf, an oil price of $50/barrel and an interest rate of 10% were used to calculate the revenue. The produced gas injection scenario uses the produced gas collected on the well site, so no transportation costs would occur. The CO_2_ cost is $1.0/Mcf with a $0.50/Mcf transportation charge (Cook 2013), while the cost associated with waterflooding is $6/barrel. The NPV for each scenario is shown in Table 4. The produced gas injection scenario presents the highest economic return, increasing the NPV by 16% compared with that of the base case. CO_2_ injection does not show advantages in the NPV calculation due to the high cost associated with purchasing and transporting CO_2_. Waterflooding shows obvious negative NPV increase in this tight gas condensate reservoir.

**Table 4:** NPV for different enhanced/improved gas methods

	Produced gas injection	CO_2_ injection	Water injection	Base case
Cumulative natural gas production, m^3^	1.60 × 10^9^	1.60 × 10^9^	1.52 × 10^9^	1.74 × 10^9^
Cumulative condensate production, m^3^	5.77 × 10^5^	5.29 × 10^5^	4.22 × 10^5^	3.78 × 10^5^
NPV (USD)	3.11 × 10^8^	2.50 × 10^8^	2.40 × 10^8^	2.69 × 10^8^

In summary, the produced gas injection has shown a considerable influence in pressure maintenance and hydrocarbon production improvement, while CO_2_ injection also leads to favorable production enhancement, yet an unfavorable NPV compared to the base case. However, such phenomenon may change if a lower cost can be achieved or the CO_2_ sequestration is considered. Water injection is not feasible in the target reservoir. The availability of the produced gas at the well site and low transportation cost indicate the produced gas injection as the best choice to enhance production in the targeted gas condensate play.

By comparing and analyzing the performance of the three EGR scenarios, it can be concluded that the main mechanism to enhance the recovery is the pressure maintenance in the tight gas condensate reservoir. For gas injection, a phase envelope change also has a favorable influence in this study. The low permeability hinders the pressure transmission and a high pressure gradient is restricted to the area near the hydraulic fractures, which limits the positive effect of viscosity and interfacial tension reduction. In addition, it is also shown that it is difficult for the injected gas to further penetrate deep into the tight matrix near fractures, leading to a large quantity of gas accumulating near the injector after gas injection. We recommend that the combination of depletion and re-injection of produced gas is the appropriate scheme for the enhanced recovery in the Montney Formation due to the good potential of production increase and lower cost.

## Sensitivity study of produced gas injection performance

The performance of the produced gas injection in the tight gas condensate reservoir can be affected by a number of parameters including reservoir properties, fracture properties, and well operational parameters. In this study, sensitivity analysis of the primary production duration, well bottom-hole pressures (BHP) and non-Darcy effects are conducted, and their effects on well production performance in the targeted tight gas condensate reservoirs are summarized.

### Effect of primary production duration

**Fig. 11:**
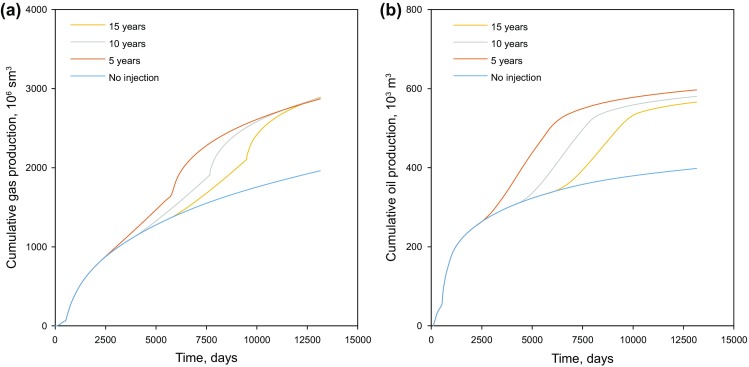
Cumulative production for scenarios with different primary production times. **a** Cumulative gas production measured under standard condition (sm^3^). **b** Cumulative condensate (oil) production

The primary production duration is a key parameter to affect the ultimate hydrocarbon recovery in the tight gas condensate reservoir. Three primary production durations of 5, 10, and 15 years are examined in the target formation to determine an appropriate length. The produced gas injection is adopted and continues for another 10 years after the primary production period, followed by reservoir depletion. During the reservoir depletion stage, the injector is converted back to a producer to recover the injected gas. The cumulative production for condensate and gas at the end of the production process is shown in Fig. 11. As seen in Fig. 11a, the cumulative gas production converges to a similar value for the three scenarios. It should be noted that for the produced gas injection scenarios part of the produced gas will be injected and reproduced from the reservoir. The injection volumes and net cumulative gas productions are listed in Table 5. The scenario of no injection still leads to the highest net gas volume although the difference is not significant (between 1.77 and 1.96 billion m^3^).

**Table 5:** Production data for different primary production duration scenarios

Scenarios	15 years	10 years	5 years	No injection
Injected gas, m^3^	1.12 × 10^9^	1.06 × 10^9^	1.00 × 10^9^	0
Net cumulative gas, m^3^	1.77 × 10^9^	1.86 × 10^9^	1.82 × 10^9^	1.96 × 10^9^
Cumulative oil, m^3^	5.66 × 10^5^	5.80 × 10^5^	5.97 × 10^5^	3.98 × 10^5^
BOE	1.43 × 10^7^	1.50 × 10^7^	1.49 × 10^7^	1.44 × 10^7^

On the other hand, the condensate production of the produced gas injection scenarios is 37%–50% higher than that of the scenario without injection. The scenario which implements produced gas injection in the 5th year yields the highest condensate production, followed by the 10-year scenario and 15-year scenario.

**Fig. 12:**
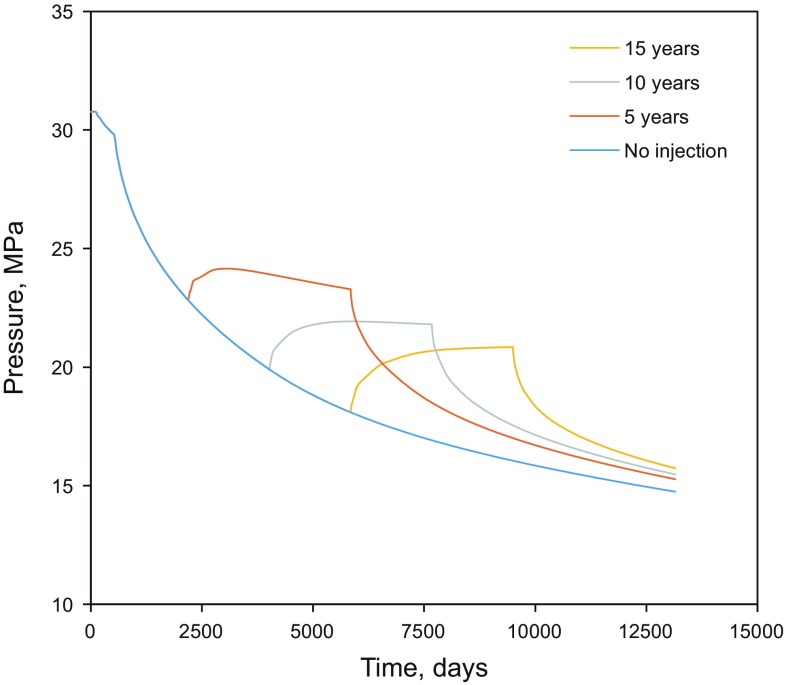
Average field pressure of different scenarios

The injected gas increases the reservoir pressure significantly and prevents the oil from being condensed out. As shown in Fig. 12, the reservoir pressure of the produced gas injection is much higher than that of the scenario without injection. The sudden increase in gas production in Fig. 11 and decrease in pressure in Fig. 12 are due to the conversion from gas injection to reservoir depletion.

### Effect of BHP

**Fig. 13:**
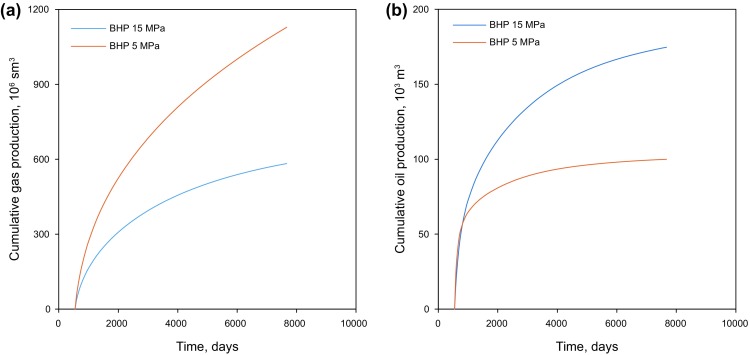
Cumulative production for scenarios with different well BHPs. **a** Cumulative gas production measured under standard condition (sm^3^). **b** Cumulative condensate (oil) production

The volume of the condensate dropped out from the gas phase is determined by the in situ pressure in the reservoir matrix pores. A lower BHP may lead to a higher gas production rate at the wellhead but also more condensate being formed in the reservoir. Such condensate liquid is typically immobile and left behind in the formation. Figure 13 depicts the cumulative condensate and gas production of two simple scenarios with BHPs of 15 and 5 MPa during 20 years of primary production.

**Fig. 14:**
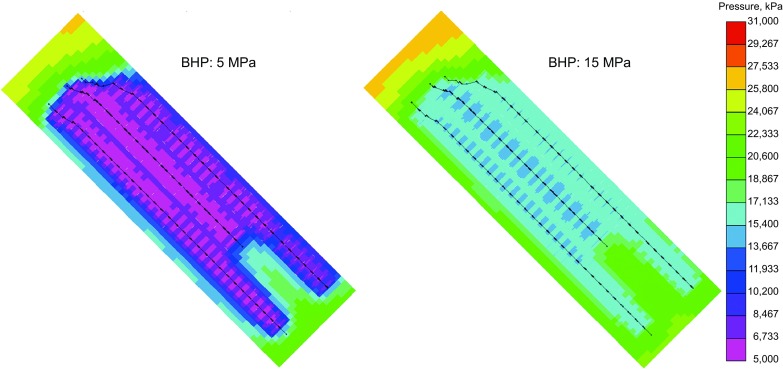
Pressure distribution of primary development after 20 years (unit kPa)

The results show that in the first couple of months, the condensate and gas production under the low BHP of 5 MPa are higher than those under the high BHP of 15 MPa, which is in accordance with the fact that a larger pressure drawdown yields a higher gas production rate. During such a short time, the low pressure at the well bottom hole has not penetrated deep into the formation, and the reservoir pressure in the formation has kept the heavy components (i.e., condensate) in the gas phase. Under such circumstances, a higher gas production rate brings more heavy components to the wellhead simultaneously, resulting in high gas and condensate production. As production proceeds, oil starts to condense from the gas phase and is left behind in the formation. The cumulative gas production of the 5 MPa scenario remains high, yet the condensate production rate is much lower than that of the 15 MPa scenario. Figure 14 depicts the pressure distribution after 20-years depletion for two scenarios with the BHPs of 5 and 15 MPa, respectively. It is shown that the pressure near the fractured horizontal wells declines far below the dew-point. Even though the driving force of BHP of 5 MPa is higher than that of BHP of 15 MPa, a large quantity of condensate oil is trapped in the reservoir due to the much lower reservoir pressure.

### Effect of hydraulic fracture conductivity

**Fig. 15:**
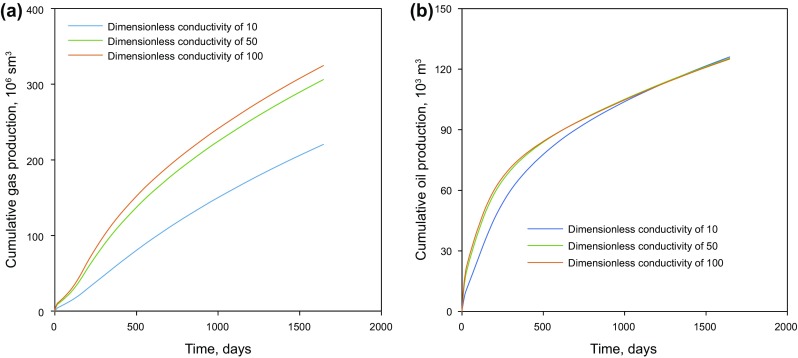
Cumulative production of the two cases with different dimensionless conductivities. **a** Cumulative gas production measured under standard condition (sm^3^). **b** Cumulative condensate (oil) production

Hydraulic fractures are the key elements in the complex fractured tight gas condensate reservoir systems and their conductivity can significantly affect the performance of the produced gas injection. Dimensionless fracture conductivity compares the ability of the hydraulic fractures to transmit the fluids to the capacity of the formation matrix to deliver the reservoir fluids into the fracture. Three scenarios, with a dimensionless conductivity of 10, 50 and 100, are used to evaluate the effects of the fracture conductivity on the well productivity during the produced gas injection process. As shown in Fig. 15, the cumulative gas production of the dimensionless conductivity of 100 is the highest, followed by that of 50, while the dimensionless conductivity of 10 scenarios displays the lowest cumulative gas production. The higher dimensionless fracture conductivity leads to a larger cumulative gas production, which corresponds to the definition of fracture conductivity in representing the fracture’s capacity to transmit fluid. In addition, the difference between the scenarios of 50 and 100 is much less than that between scenarios of 10 and 50.

The cumulative condensate production curves, however, demonstrate different behavior. At the primary production period of the first 500 days, the dimensionless conductivity of 100 and 50 are close in showing a larger oil production, while the dimensionless conductivity of 10 has the lowest oil production. During 500–1000 days, the oil production rate of dimensionless conductivity of 100 and 50 tends to grow more slowly than that of the dimensionless conductivity of 10, while beyond Day 1000, the cumulative oil production of the dimensionless conductivity of 10 catches up with the other two scenarios. At the end of 1600 days, the oil production of the conductivity of 10 exceeds the other two scenarios.

In the two higher dimensionless conductivities, the fracture transmission ability is much higher, which means that the reservoir pressure drops faster. As production proceeds, the reservoir pressure declines down to the dew-point pressure; liquids begin to condensate near the wellbore and block the gas from flowing to the wellbore. The condensate liquid remains unrecovered in the reservoir, decreasing the condensate production rate at the wellhead significantly. Thus, in the scenario of the dimensionless conductivity of 10, the pressure drops more slowly, resulting in a higher oil production later in the production period.

### Effect of non-Darcy flow in hydraulic fractures

In the tight gas condensate reservoirs, non-Darcy flow behavior could appear when the gas flow rate exceeds the limit for Darcy’s equation application scope, and results in an additional pressure loss in hydraulic fractures. The Reynolds number and the Forchheimer number are the two key criteria to identify the non-Darcy flow. In this study, the Forchheimer equation (Eq. 3) is utilized to study the non-Darcy effect in the gas condensate reservoirs (Rubin 2010; Yu et al. 2014).

3 $$- \nabla p = \frac{\mu }{k}v + \beta \rho v^{2}$$

where *μ* is viscosity, *v* is velocity, *k* is the hydraulic fracture permeability, *β* is the non-Darcy Beta factor and *ρ* is the density of the phase.

**Fig. 16:**
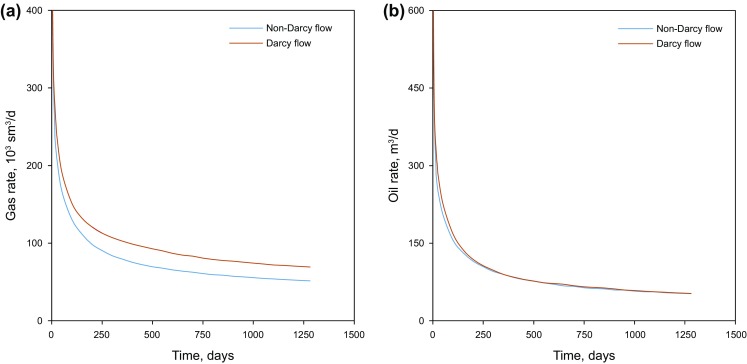
Comparison of production rates for the scenarios with Darcy and non-Darcy flow. **a** Gas rate of Darcy and non-Darcy flow measured under standard condition (sm^3^/d). **b** Condensate (oil) rate of Darcy and non-Darcy flow

Figure 16 depicts the well production performance with two scenarios; considering and ignoring the non-Darcy flow effects. It can be seen that considerable differences exist between the gas production rates with the two scenarios. Ignoring the non-Darcy flow effects can over-estimate the gas flow rate by 40% after the rate curve stabilizes in the first 3 months. Figure 16b demonstrates that the condensate rates are almost unchanged due to a low condensate flow rate in the fractures compared to the gas rate.

## Conclusions

Production performance of the produced gas injection, CO_2_ injection and water injection is investigated in this study. The following conclusions are drawn:

1. Produced gas injection demonstrates better performance in increasing the cumulative gas and condensate (oil) production in the targeted tight gas condensate reservoir. The cumulative condensate production is 52.7% higher than that of the base case where no fluid is injected. The NPV calculation also indicates that produced gas is the most economical method, owing to higher production rates, the easy access of the injection gas resource and no gas separation charge.
2. Both the cumulative condensate production and BOE are improved by the CO_2_ injection; however, its NPV is lower compared to those from the base case. Such conclusion may not be valid for a different tight gas condensate reservoir as the cost to purchase and transport CO_2_ is different for each project. Water injection is the worst option to enhance production or maintain reservoir pressure due to the poor injection ability in the tight gas condensate reservoir.
3. A sensitivity study shows that a short primary production period (5 years in this study) delivers a better performance compared to the remaining scenarios. A low BHP leads to a higher pressure difference and, thus, a higher gas production; however, the reservoir pressure rapidly drops below the dew-point pressure, leading to a large amount of liquid condensation, which significantly decreases condensate production in the long term.
4. A higher hydraulic fracture conductivity is beneficial to both cumulative gas and cumulative oil production during the initial production period. As the reservoir pressure drops below the dew-point pressure, however, significant quantities of condensate oil will emerge from the gas phase, blocking the gas flow to the wellbore and reducing oil production. In addition, non-Darcy flow behavior exists with a high gas rate, while it does not have a noticeable effect on the condensate production. This is because that the condensate flow rate in the fractures is much lower than that of the gas.
